# Upregulation of Chemoresistance by Mg^2+^ Deficiency through Elevation of ATP Binding Cassette Subfamily B Member 1 Expression in Human Lung Adenocarcinoma A549 Cells

**DOI:** 10.3390/cells10051179

**Published:** 2021-05-12

**Authors:** Saki Onuma, Aya Manabe, Yuta Yoshino, Toshiyuki Matsunaga, Tomohiro Asai, Akira Ikari

**Affiliations:** 1 Laboratory of Biochemistry, Department of Biopharmaceutical Sciences, Gifu Pharmaceutical University, Gifu 501-1196, Japan; 165019@gifu-pu.ac.jp (S.O.); 135072@gifu-pu.ac.jp (A.M.); yoshino-yu@gifu-pu.ac.jp (Y.Y.); 2 Education Center of Green Pharmaceutical Sciences, Gifu Pharmaceutical University, Gifu 502-8585, Japan; matsunagat@gifu-pu.ac.jp; 3 Department of Medical Biochemistry, School of Pharmaceutical Sciences, University of Shizuoka, Shizuoka 422-8526, Japan; asai@u-shizuoka-ken.ac.jp

**Keywords:** lung adenocarcinoma, chemoresistance, ABCB1, hypomagnesemia

## Abstract

Several anticancer drugs including cisplatin (CDDP) induce hypomagnesemia. However, it remains fully uncertain whether Mg^2+^ deficiency affects chemosensitivity of cancer cells. Here, we investigated the effect of low Mg^2+^ concentration (LM) on proliferation and chemosensitivity using human lung adenocarcinoma A549 cells. Cell proliferation was reduced by continuous culture with LM accompanied with the elevation of G1 phase proportion. The amounts of reactive oxygen species (ROS) and stress makers such as phosphorylated-ataxia telangiectasia mutated and phosphorylated-p53 were increased by LM. Cell injury was dose-dependently increased by anticancer drugs such as CDDP and doxorubicin (DXR), which were suppressed by LM. Similar results were obtained by roscovitine, a cell cycle inhibitor. These results suggest that LM induces chemoresistance mediated by ROS production and G1 arrest. The mRNA and protein levels of ATP binding cassette subfamily B member 1 (ABCB1) were increased by LM and roscovitine. The LM-induced elevation of ABCB1 and nuclear p38 expression was suppressed by SB203580, a p38 MAPK inhibitor. PSC833, an ABCB1 inhibitor, and SB203580 rescued the sensitivity to anticancer drugs. In addition, cancer stemness properties were suppressed by SB203580. We suggest that Mg^2+^ deficiency reduces the chemotherapy sensitivity of A549 cells, although it suppresses cell proliferation.

## 1. Introduction

Magnesium (Mg^2+^) is the fourth most abundant cation in the body and an essential electrolyte with various physiological functions. Serum Mg^2+^ concentration is tightly regulated within a narrow range (0.7–1.0 mM) mediated by the absorption and reabsorption pathways in the intestine and kidney, respectively [1]. Mg^2+^ deficiency can be induced by poor oral intake, increased renal loss, and chronic diarrhea. In addition, loss-of-function mutations in the human *transient receptor potential melastatin 6 (TRPM6)* cause hereditary hypomagnesemia and secondary hypocalcemia [2]. In a nested case-control study, high and low serum Mg^2+^ concentrations are associated with an increased incident risk of cancer [3]. Furthermore, lower expression level of TRPM6 is associated with poor prognosis in patients with colorectal, breast gastric, and lung cancers [4]. Some anticancer drugs, including cisplatin (CDDP), a platinum based anticancer drug, and cetuximab and panitumumab, chimeric and fully human monoclonal antibodies to the epidermal growth factor receptor (EGFR), often cause a side effect of hypomagnesemia [5]. Cetuximab downregulates TRPM6-mediated Mg^2+^ influx by interfering with EGF signaling [6]. In addition, anti-EGFR agents induce kidney and cardiac disorders [7,8]. However, the effect of hypomagnesemia on proliferation and anticancer activity in cancer cells has not been fully understood.

Mg^2+^ is a cofactor for more than 300 enzymes involved in ATP-dependent biochemical processes including cell proliferation, cell cycle regulation, and protein synthesis [9]. The cell cycle is divided into four phases: gap 1 (G1), synthesis (S), G2, and mitosis (M). G1 is the gap phase during which cells prepare for the process of DNA replication [10]. The proper progression of cell cycle is checked in G1-to-S and G2-to-M transition processes. These processes are controlled by cyclin-dependent kinases (CDKs), which are complexed with a regulatory subunit (cyclin). DNA damage activates the tumor suppresser protein p53 and CDK inhibitor p21, resulting in cell cycle arrest at G1 phase by inhibition of CDK activity and DNA replication. Ataxia telangiectasia mutated (ATM) regulates DNA damage responses caused by the activation of important substrates involved in DNA repair and cell cycle control. Several factors including p53 are present in the downstream of ATM signaling [11], and the activation of ATM/p53 pathway causes the inhibition of cell proliferation. Genetic or pharmacologic inhibition of TRPM7, which may form heteromeric channels with TRPM6, have been reported to reduce proliferation of lung [12], breast [13], and gastric cancer cells [14]. On the other hand, the correlation between chemoresistance and cell cycle arrest has not completely elucidated yet.

The chemoresistance is one of the major limitations of cancer therapy [15,16]. The ATP-binding cassette (ABC) transporter family contributes to the acquisition of chemoresistance in several malignancies [17]. Human ABC transporters are divided into seven (A to G) subfamilies [18]. Among them, ABCB1 is highly expressed in patients with lung adenocarcinoma and associated with poor survival [19]. ABCB1 inhibitors can overcome resistance to CDDP [20], doxorubicin (DXR, an anthracycline antibiotic) [21], and paclitaxel (a taxane-derived chemotherapeutic agent) [22], in human lung adenocarcinoma cells. Recently, the formation of tumor microenvironment by extracellular matrix, cancer cells, and non-cancerous cells has been reported to be involved in the chemoresistance [23]. The inside cells of microenvironment are commonly exposed to hypoxic, oxidative, hypoglycemic, and acidosis stress conditions. Hypoxia-inducible factor-1 (HIF-1) and nuclear factor-erythroid 2 related factor 2 (Nrf2) play important roles in the hypoxic and oxidative stress responses, respectively [24]. In addition, several intracellular signaling factors including Jun N-terminal kinase (JNK) and p38 mitogen-activated protein kinase (MAPK) are activated in the microenvironment [25]. The expression levels of phosphorylated (p)-p38 and p-JNK in human non-small cell lung cancer (NSCLC) tissues are higher than those in normal tissues [26]. The improvement of stress conditions or inhibition of stress response signals may be useful to prevent the tumor progression.

In the present study, we found that CDDP- and DXR-induced injuries are suppressed by culturing A549 cells, a cell line derived from human lung adenocarcinoma, with low Mg^2+^ concentration (LM). The mRNA and protein expressions of ABC transporters were investigated by real-time polymerase chain reaction (PCR) and Western blotting analyses, respectively. The production of cytosolic and mitochondrial reactive oxygen species (ROS) was monitored using fluorescent indicators. Our results indicate that Mg^2+^ deficiency may attenuate the chemosensitivity of A549 cells against anticancer drugs mediated through the elevation of ABCB1 expression.

## 2. Materials and Methods

### 2.1. Cell Culture

A549 (RCB0098), PC-3 (JCRB0077), and H1299 (CRL-5803) cells, derived from human lung adenocarcinoma, were purchased from the RIKEN BRC through the National Bio-Resource Project of the MEXT (Ibaraki, Japan), JCRB Cell Bank (Osaka, Japan), and ATCC (Rockville, MD, USA), respectively. The cells were continuously cultured in the Dulbecco’s modified Eagle’s medium (DMEM, Fujifilm Wako Pure Chemical, Osaka, Japan) containing normal concentration of Mg^2+^ (0.8 mM Mg^2+^, NM) or LM (0.5 mM Mg^2+^) supplanted with 5% fetal calf serum, 0.07 mg/mL of penicillin-G potassium, and 0.14 mg/mL of streptomycin sulfate, as described previously [27]. LM medium was prepared according to the composition of normal DMEM without Mg^2+^ concentration. In the assay of chemoresistance, the cells were incubated with anticancer drugs for 24 h in the absence of fetal bovine serum. The cell injury was examined using the Premix WST-1 Cell Proliferation Assay System (Takara-Bio, Shiga, Japan) in accordance with the manufacturer’s instructions. After subtracting background values, the relative cell injury was calculated with the formula: relative cell injury (%) = (1 – OD values in anticancer drug-treated cells/OD values in untreated cells) × 100.

### 2.2. Measurement of [Mg^2+^]_i_

A549 cells (5 × 10^3^/well) were cultured on 96-well black/clear bottom plate, TC Surface (Thermo Fisher Scientific, San Jose, CA, USA) for 3 days. Then, the cells were incubated with Hank’s balanced salt solution (HBSS) containing Mag-fura-2/AM (AAT Bioquest, Sunnyvale, CA, USA) for 30 min at 37 °C. After washing twice with dye-free HBSS, the plate was set on a fluorescence microplate reader (Infinite F200 PRO, Tecan, Mannedorf, Switzerland). The fluorescence intensities of Mag-fura-2 were monitored at 340/535 and 430/535 nm. [Mg^2+^]_i_ was calculated from the 340/380 nm ratio in accordance with the formula of Grynkiewicz et al. [28].

### 2.3. Reverse Transcription and Quantitative Real-Time Polymerase Chain Reaction (PCR)

Cells (1 × 10^5^/dish) were cultured on a 60-mm EasYDish, TC Surface for 3 days. Isolation of total RNA, reverse transcription, and quantitative real-time PCR were performed as described previously [29]. The specific primer pairs against human Mg^2+^ transporters including TRPM6, TRPM7, CNNM2, MagT1, SLC41A1, and MRS2 are listed in Table 1.

### 2.4. Flow Cytometry

In the cell cycle analysis, A549 cells (1 × 10^5^/dish) were cultured on a 60-mm EasYDish, TC Surface (Thermo Fisher Scientific) for 2 days. Then, the cells were stained with a Muse Cell Cycle Kit (Luminex, Austin, TX, USA) in accordance with the manufacturer’s specifications. In the CSLCs analysis, the cells were stained with phycoerythrin (PE)-conjugated anti-CD133 antibody (B262394, clone 7, Biolegend, San Diego, CA, USA). The threshold was given by unstained cells. The percentage of cells in the G0/G1, S, and G2/M phases of cell cycle, and CD133-positive cells were monitored using a Muse Cell Analyzer (Luminex).

### 2.5. Sodium Dodecyl Sulfate-Polyacrylamide Gel Electrophoresis (SDS-PAGE) and Western Blotting

Cells (1 × 10^5^/dish) were cultured on a 60-mm EasYDish, TC Surface for 3 days. The preparation of cell lysates, SDS-PAGE, and Western blotting were performed as described previously [27]. Nuclear protein was isolated using NE-PER nuclear and cytoplasmic extraction reagents (Thermo Fisher Scientific). Primary antibodies used in Western blotting are listed in Table 2. The blots were scanned using a C-DiGit Blot Scanner (LI-COR Biotechnology, Lincoln, NE, USA). Band density was quantified using ImageJ software (National Institute of Health, Bethesda, MD, USA). β-Actin or nucleoporin p62 was used as internal loading controls.

### 2.6. Oxidative DNA Damage and ROS Production

Cells (5 × 10^3^/well) were cultured on a 96-well black/clear bottom plate, TC Surface for 3 days. In the ROS production assay, the cells were incubated with HBSS containing Hoechst33342 (a nuclear marker) plus 2’,7’-dichlorodihydrofluorescein diacetate (H_2_DCFDA, Thermo Fisher Scientific) or MitoROS 580 (AAT Bioquest, Sunnyvale, CA, USA) at 37 °C for 30 min. After washing twice with dye-free HBSS, the fluorescence images were observed using a fluorescence microplate reader. The fluorescence intensities of H_2_DCF and MitoROS were corrected by Hoechst33342 and represented as percentage of NM. Oxidative DNA damage was examined using a DNA damage (8-oxo-dG) ELISA kit (StressMarq Biosciences, Victoria, BC, Canada). The absorbance at 450 nm was measured by an iMark microplate reader (Bio-Rad Laboratories, Richmond, CA, USA). The content of 8-oxo-dG was calculated using calibration curve.

### 2.7. Statistical Analysis

Results are presented as means ± S.E.M. Differences between groups were analyzed by one-way analysis of variance, and corrections for multiple comparison were made using Tukey’s multiple comparison test. Comparisons between two groups were made using a Student’s *t*-test. Statistical analyses were performed using KaleidaGraph version 4.5.1 software (Synergy Software, Reading, PA, USA). Significant differences were assumed at *p* < 0.05.

## 3. Results

### 3.1. Inhibition of Cell Proliferation by Culturing in LM Medium

**Figure 1:**
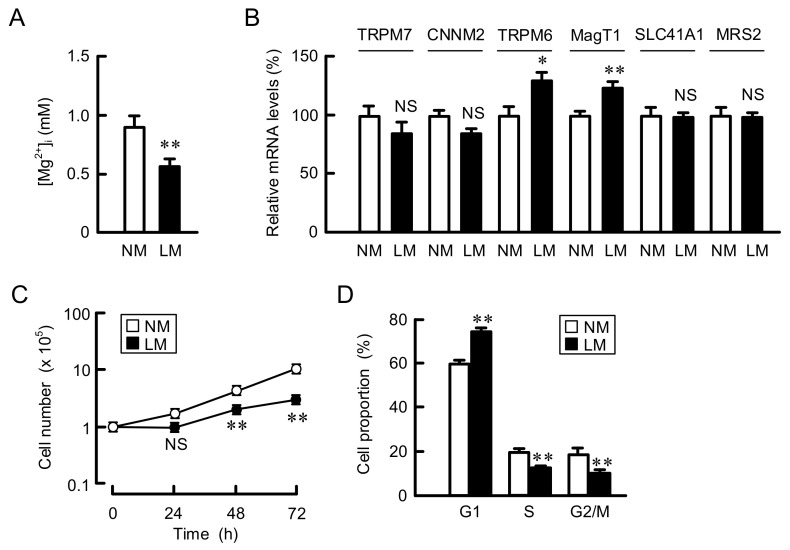
Inhibition of proliferation by LM in A549 cells. A549 cells were continuously cultured in the media containing normal 0.8 mM Mg^2+^ (NM) or 0.5 mM Mg^2+^ (LM). (**A**) [Mg^2+^]_i_ was measured using Mag-fura 2. (**B**) The mRNA levels of Mg^2+^ transporters were examined by real-time PCR and represented in percentage to NM. (**C**) Cell number was counted at the time indicated. (**D**) The proportion of G1, S, and G2/M was measured using Muse Cell Analyzer. n = 3–4. ** *p* < 0.01 and * *p* < 0.05 compared with NM. NS *p* > 0.05.

A549 cells were continuously cultured in the media containing normal concentration of Mg^2+^ (0.8 mM Mg^2+^, NM) or LM (0.5 mM Mg^2+^). Intracellular free Mg^2+^ concentration ([Mg^2+^]_i_) was significantly decreased by LM (Figure 1A). The mRNA levels of Mg^2+^ transporters, transient receptor potential melastatin 6 (TRPM6), and magnesium transporter 1 (MagT1) were increased by LM (Figure 1B). In contrast, those of other Mg^2+^ transporters including TRPM7, cyclin M2 (CNNM2 known as ancient conserved domain protein), solute carrier family 41 member A1 (SLC41A1), and mitochondrial RNA splicing 2 (MRS2) were not significantly changed. Cell proliferation was reduced by LM, whose effects were significant at 48 and 72 h (Figure 1C). The doubling times of cells cultured in NM and LM were 21.1 and 44.9 h, respectively. The proportion of cells in the G1 phase was increased by LM, while the proportion of cells in S and G2/M phases was decreased (Figure 1D). These results indicate that the G1-S cell cycle progression may be suppressed by LM.

### 3.2. Increase in the Expression of Negative Cell Cycle Regulators by LM

**Figure 2:**
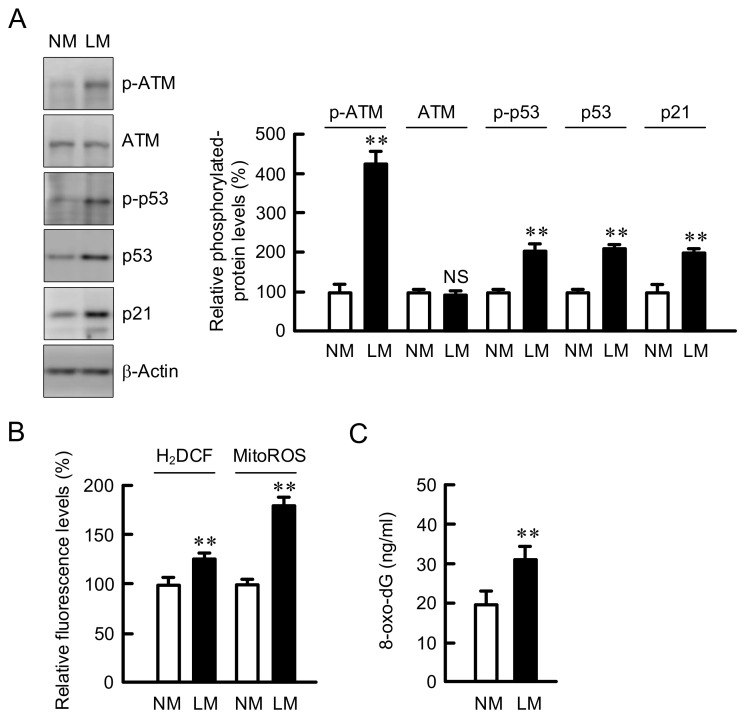
Effects of LM on cell cycle regulators, ROS production, and oxidative-DNA damage. A549 cells were continuously cultured in the media containing NM or LM. (**A**) Western blotting was performed using anti-p-ATM, anti-ATM, anti-p-p53, anti-p53, anti-p21, and anti-β-actin antibodies. The expression levels of these proteins were corrected by β-actin and are represented in percentage to NM. (**B**) The cells were incubated with Hoechst33342 plus H_2_DCFAM or MitoROS for 30 min. The relative fluorescence intensities of H_2_DCF and MitoROS were represented as percentage of NM. (**C**) The content of 8-oxo-dG was examined using DNA damage (8-oxo-dG) ELISA kit and calculated using calibration curve. n = 3–8. ** *p* < 0.01 compared with NM. NS, *p* > 0.05.

The G1-S cell cycle progression is upregulated by CDK2, whose function is inhibited by ATM/p53/p21 signaling pathway [30]. The expression levels of p-ATM, p-p53, p53, and p21 were increased by LM without affecting total amount of ATM (Figure 2A). The cell cycle arrest is affected by ROS production [31]. The levels of cytosolic and mitochondrial ROS were increased by LM (Figure 2B). In addition, the level of 8-oxo-dG, a marker for oxidative DNA damage, was increased by LM (Figure 2C). These results indicate that LM may cause cell cycle arrest mediated by the chronic elevation of ROS production and oxidative DNA damage.

### 3.3. Decrease in Chemosensitivity of Anticancer Drugs by LM

**Figure 3:**
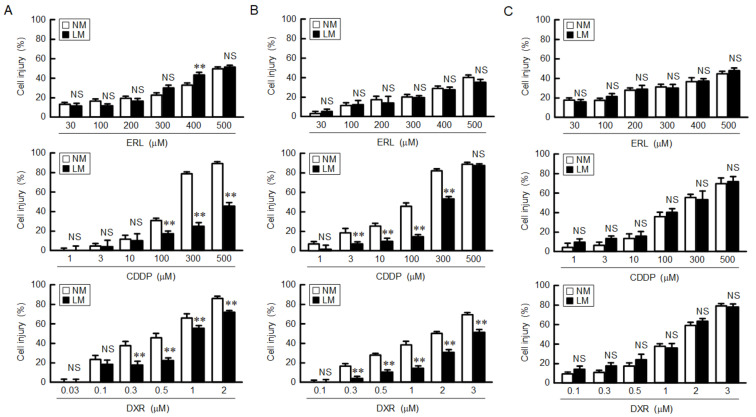
Reduction of anticancer drug-induced cell injury by LM. A549 (**A**), PC-3 (**B**), and H1299 (**C**) cells were continuously cultured in the media containing NM or LM. The cells were incubated with ERL, CDDP, and DXR at the concentration indicated for 24 h. Cell injury was measured using the Premix WST-1 Cell Proliferation Assay System. n = 5–8. ** *p* < 0.01 compared with NM. NS, *p* > 0.05.

**Figure 4:**
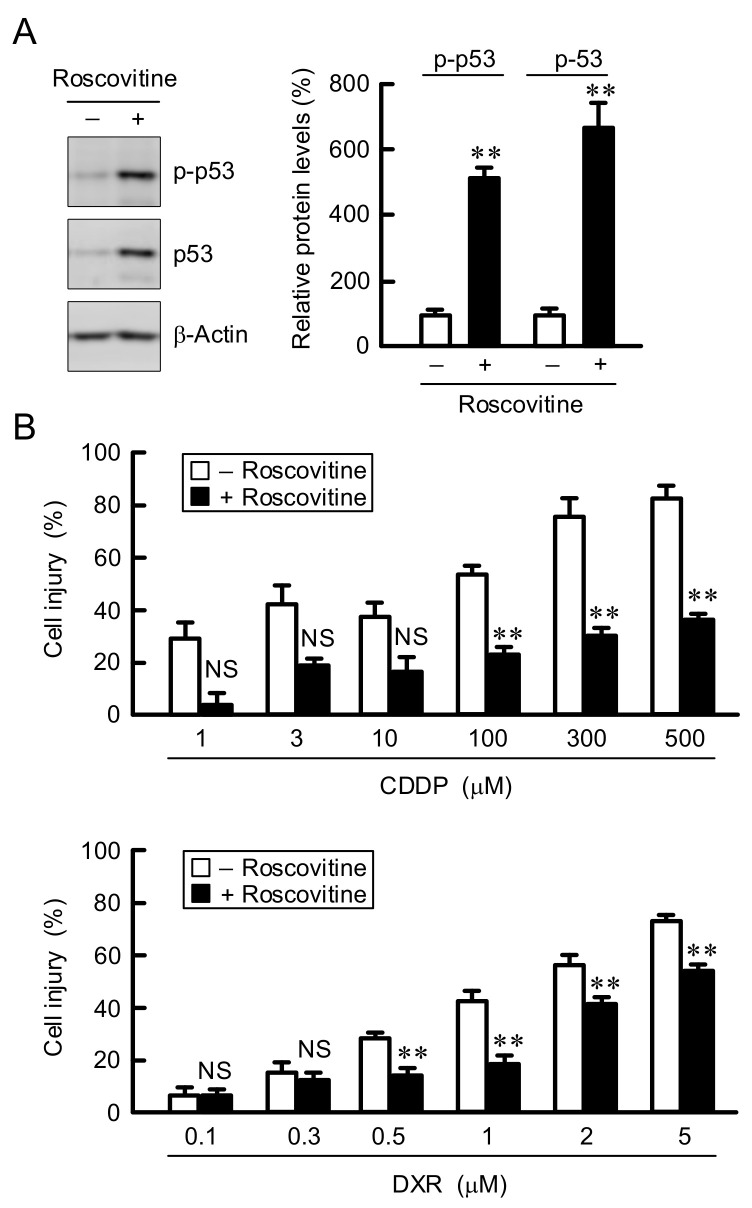
Reduction of anticancer drug-induced cell injury by roscovitine. (**A**) A549 cells were incubated in the absence and presence of 10 μM of roscovitine for 3 h. Western blotting was performed using anti-p-p53, anti-p53, and anti-β-actin antibodies. The expression levels of p-p53 and p53 were corrected by β-actin. The protein levels are represented in percentage to the cells without roscovitine. (**B**) After treatment with 10 μM roscovitine for 24 h, the cells were incubated in the absence and presence of CDDP or DXR at the concentration indicated for 24 h. Cell injury was measured using the Premix WST-1 Cell Proliferation Assay System. n = 3–8. ** *p* < 0.01 compared with -roscovitine. NS, *p* > 0.05.

Cell injury was dose-dependently increased by the treatments with erlotinib (ERL), CDDP, and DXR in A549 cells (Figure 3A). The CDDP- and DXR-induced injuries were suppressed by LM, whereas the ERL-induced injuries were not. Similarly, the CDDP- and DXR-induced injuries were suppressed by LM in human lung adenocarcinoma-derived PC-3 cells (Figure 3B). In contrast, the anticancer-induced injuries were not suppressed by LM in H1299 cells, which lack endogenous p53. These results indicate that LM may induce chemoresistance in the p53-expressing adenocarcinoma cells. As shown above, the G1-S cell cycle progression was inhibited by LM. Therefore, we investigated the effect of roscovitine, a potent and selective inhibitor of CDKs, on chemosensitivity. The levels of p-p53 and p53 were significantly increased by roscovitine in A549 cells (Figure 4A). The CDDP- and DXR-induced cell injuries were suppressed by roscovitine (Figure 4B). These results coincided with those of LM.

### 3.4. Elevation of ABCB1 Expression by LM and Roscovitine

**Figure 5:**
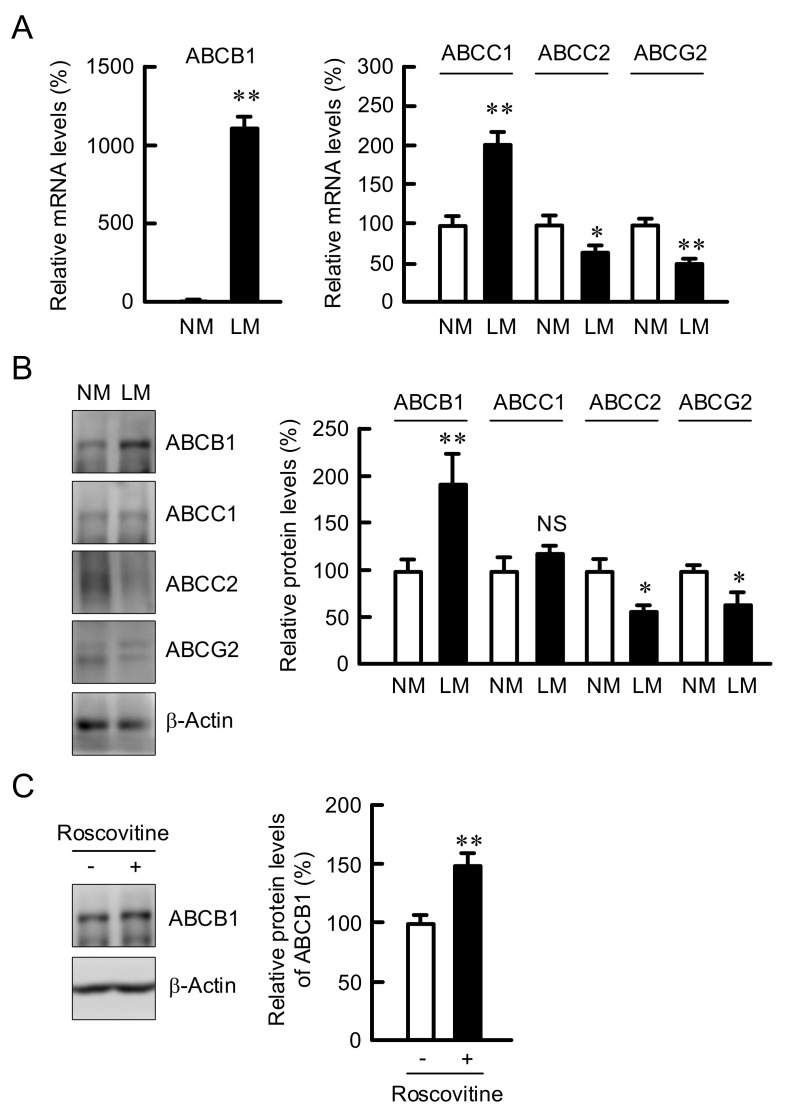
Increase in the expression of ABCB1 by LM and roscovitine. (**A**) A549 cells were continuously cultured in the media containing NM or LM. Real-time PCR was performed using primer pairs for ABCB1, ABCC1, ABCC2, ABCG2, and β-actin. The mRNA levels are represented in percentage to NM. (**B**) Western blotting was performed using anti-ABCB1, anti-ABCC1, anti-ABCC2, anti-ABCG2, and anti-β-actin antibodies. The protein levels are represented in percentage to NM. (**C**) The cells cultured in the NM medium were incubated in the absence and presence of 10 μM roscovitine for 24 h. After Western blotting with anti-ABCB1 and anti-β-actin antibodies, the protein levels are represented in percentage to the cells without roscovitine. n = 3–4. ** *p* < 0.01 and * *p* < 0.05 compared with NM or -roscovitine. NS, *p* > 0.05.

ABC transporters including ABCB1, ABCC1, ABCC2, and ABCG2 are involved in the reduction of chemosensitivity in cancer cells [17]. The mRNA levels of ABCB1 and ABCC1 were elevated by LM; in particular, ABCB1 expression increased over 100-fold (Figure 5A). In contrast, the mRNA levels of ABCC2 and ABCG2 were decreased by LM. Similarly, the protein levels of ABC transportes were changed by LM in Western blotting analysis (Figure 5B). The protein level of ABCB1 was also increased by roscovitine (Figure 5C). These results indicate that the expression of ABCB1 may be controlled by cell cycle regulators.

### 3.5. Involvement of p38 in the Elevation of ABCB1 Expression by LM

**Figure 6:**
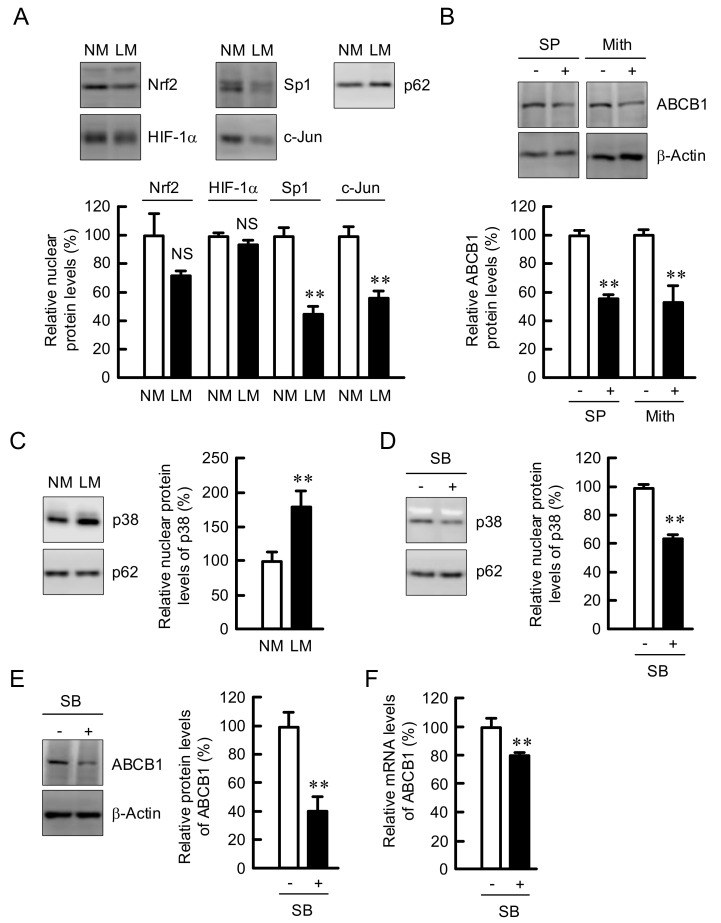
Decrease in the expression of ABCB1 by SB203580. (**A**,**C**) A549 cells were continuously cultured in the media containing NM or LM. The nuclear fraction was applied on SDS-PAGE. Western blotting was performed using anti-Nrf2, anti-HIF-1α, anti-Sp1, anti-c-Jun, anti-p38, and anti-nucleoporin p62 (p62) antibodies. The nuclear protein levels are represented in percentage to NM. (**B**) A549 cells cultured in the NM medium were incubated in the absence and presence of 10 μM SP600125 (SP) or 400 nM mithramycin (Mith). The protein levels are represented in percentage to the cells without SP or Mith. (**D**–**E**) The cells cultured in LM were incubated in the absence and presence of 10 μM SB203580 (SB) for 3 (**D**), 24 (**E**), or 6 h (**F**). (**D**) Western blotting of the nuclear fraction was performed using anti-p38 and anti-nucleoporin p62 antibodies. (**E**) Western blotting of the cell lysates was performed using anti-ABCB1 and anti-β-actin antibodies. (**F**) Real-time PCR was performed using primer pairs for ABCB1 and β-actin. The protein and mRNA levels are represented in percentage to the cells without SB. n = 3-4. ** *p* < 0.01 compared with NM, -SP, -Mith, or -SB. NS, *p* > 0.05.

To clarify the regulatory mechanism of ABCB1 expression, we investigated the nuclear levels of transcriptional regulatory factors of ABCB1 [32,33]. The nuclear levels of Sp1 and c-Jun were decreased by LM, whereas those of Nrf2 and HIF-1*α* were unchanged (Figure 6A). The protein level of ABCB1 was decreased by SP600125, a c-Jun inhibitor, and mithramycin, an Sp1 inhibitor, in the cells cultured with NM medium (Figure 6B), indicating that neither c-Jun nor Sp1 may be involved in the elevation of ABCB1 expression by LM. The nuclear level of p38 was increased by LM (Figure 6C). The levels of ABCB1 and nuclear p38 were significantly decreased by SB203580, a p38 MAPK inhibitor, in the cells cultured in LM medium (Figure 6D), indicating that p38 MAPK may be involved in the LM medium-induced elevation of ABCB1 expression. The mRNA level of ABCB1 was decreased by LM, but the effect was smaller than that in Western blotting analysis (Figure 6E,F).

### 3.6. Effect of LM on Chemoresistance

**Figure 7:**
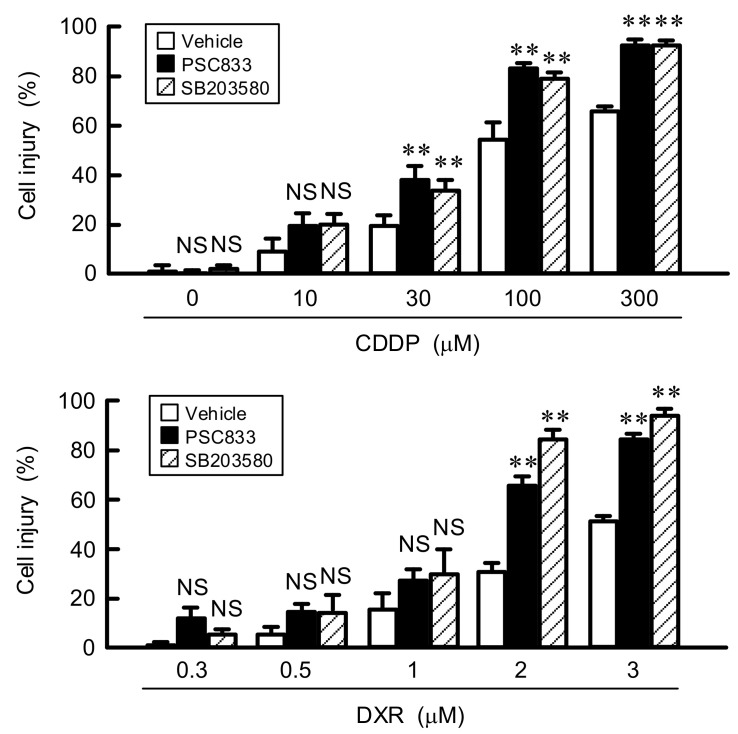
Rescue of LM-induced chemoresistance by PSC833 and SB203580. A549 cells cultured with LM were pre-treated with vehicle (dimethyl sulfoxide), 10 μM of PSC833, or 10 μM of SB203580 for 24 h. Then, the cells were incubated with CDDP and DXR at the concentration indicated for 24 h. Cell injury was measured using the Premix WST-1 Cell Proliferation Assay System. n = 6–8. ** *p* < 0.01 compared with vehicle. NS, *p* > 0.05.

To clarify the involvement of ABCB1 in chemoresistance by LM, we investigated the effects of PSC833, a substrate and inhibitor of ABCB1, and SB203580 on cell injury. As shown above, the expression of ABCB1 is decreased by SB203580. The CDPP- and DXR-induced elevation of cell injury was significantly exaggerated by PSC833 and SB203580 (Figure 7). These results indicate that ABCB1 induced by LM may be involved in the acquisition of resistance against anticancer drugs.

### 3.7. Effect of LM on the Proportion of Cancer Stem Like Cells (CSLCs)

**Figure 8:**
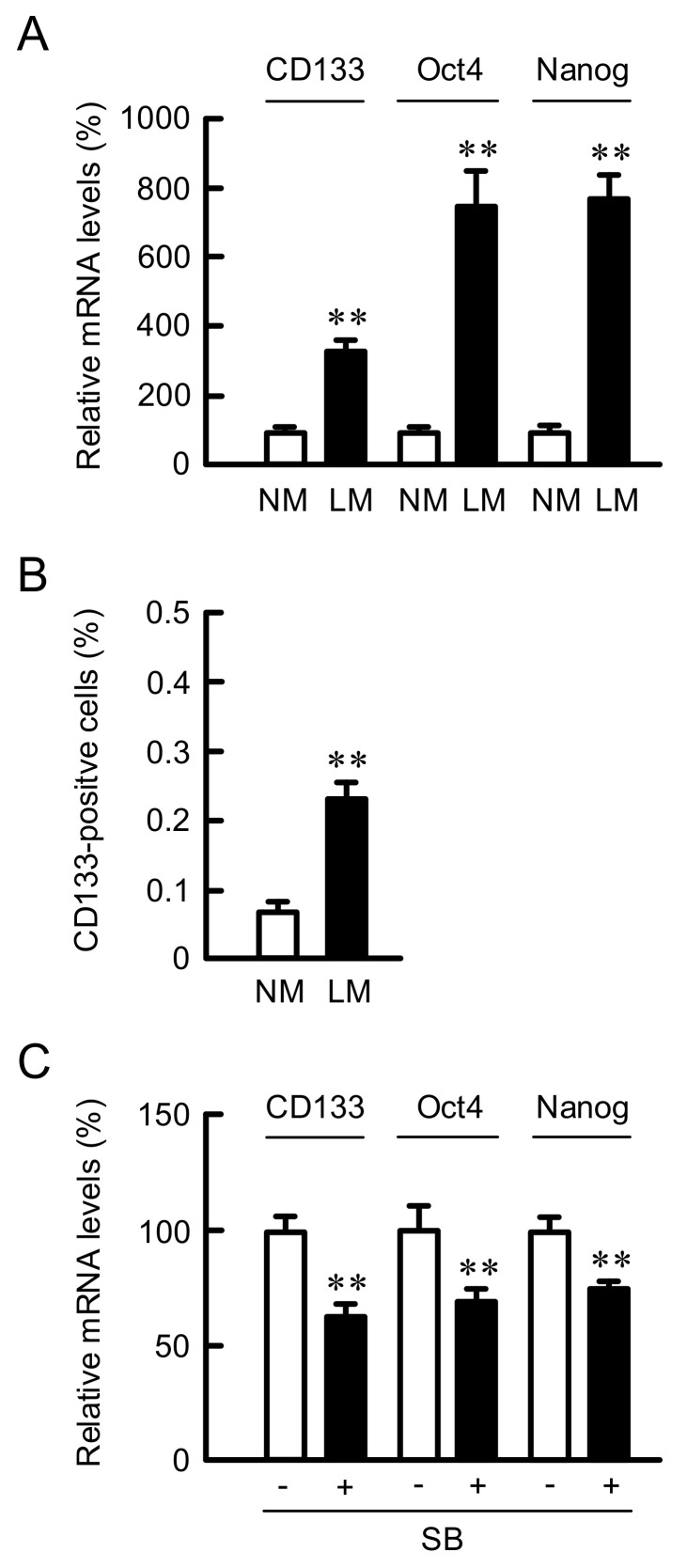
Increase in proportion of CSLCs by LM. A549 cells were continuously cultured in the media containing NM or LM. (**A**) Real-time PCR was performed using primer pairs for CD133, Oct4, Nanog, and β-actin. The mRNA levels are represented in percentage to LM. (**B**) The cells were stained with PE-conjugated anti-CD133 antibody. The percentages of CD133-positive cells were measured using a Muse Cell Analyzer. (**C**) A549 cells cultured with LM were incubated in the absence and presence of 10 μM SB203580 (SB) for 6 h. Real-time PCR was performed using primer pairs for CD133, Oct4, Nanog, and β-actin. The mRNA levels are represented in percentage to the cells without SB. n = 4. ** *p* < 0.01 compared with NM.

**Figure 9:**
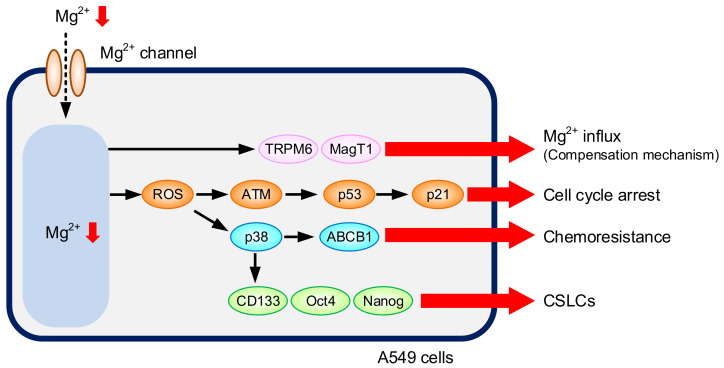
A putative model of LM-induced responses. [Mg^2+^]_i_ is reduced in the cells continuously cultured with LM. LM induces G1-S cell cycle arrest mediated through the elevation of p-ATM, p-p53, and p21. The mRNA levels of TRPM6 and MagT1 are increased by LM. LM increases the mRNA and protein levels of ABCB1 mediated by the activation of p38, leading to the acquisition of chemoresistance. LM increases CD133-positive cells, indicating the elevation of proportion of CSLCs.

CSLCs in NSCLC are identified as a rare sub-population of undifferentiated CD133-positive cells [34]. In addition, CSLCs express stem cell markers including Octamer-binding transcription factor 4 (Oct4), SRY (sex-determining region Y)-box 2 (Sox2), and Nanog transcription factors [35]. The mRNA levels of CD133, Oct4, and Nanog were increased by LM (Figure 8A). These results indicate that the proportion of CSLCs may be increased by LM. To clarify this hypothesis, we carried out flow cytometric analysis. The proportion of CD133-positive cells was increased by LM (Figure 8B). The mRNA levels of CD133, Oct4, and Nanog in the cells cultured with LM were significantly decreased by SB203580 (Figure 8C). A putative model of LM-induced responses is shown in Figure 9.

## 4. Discussion

TRPM6 is exclusively expressed in the intestine and distal collecting tubule of the kidney, and plays roles on the regulation of absorption and reabsorption of Mg^2+^ [36]. Similarly, CNNM2 and CNNM4 may be involved in the transport of Mg^2+^ in the intestine and kidney, respectively. On the other hand, SLC41A1, TRPM7, MagT1, and MRS2 are ubiquitously expressed in the mammalian cells and play a role on the regulation of intracellular Mg^2+^ homeostasis. The expression and activity of Mg^2+^ transporters are tightly regulated in order to maintain normal physiological functions. We found that the culture of A549 cells with LM medium decreases [Mg^2+^]_i_ and induces the elevation of mRNA levels of TRPM6 and MagT1 (Figure 1A,B). In contrast, those of TRPM7, CNNM2, SLC41A1, and MRS2 were not significantly changed. These results suggest that the expression of TRPM6 and MagT1 may be compensatory increased by LM. Qin et al. [4] reported that the reduction of TRPM6 expression, which may induce the reduction of [Mg^2+^]_i_, is associated with lower overall survival rates in the patients with lung adenocarcinoma with a 10-year follow-up using Kaplan–Meier plotter analysis. Hypomagnesemia is often caused by the administration of anticancer drugs including CDDP and anti-EGFR antibody [5]. This raises a possibility that hypomagnesemia interferes the ability of anticancer drugs to inhibit proliferation and induce cell death. However, little is known about whether hypomagnesemia can affect cancer chemotherapy.

The rate of cell proliferation in LM medium was lower than that in NM medium in A549 cells (Figure 1C). The proportion in G1 phase was increased by LM (Figure 1D), suggesting that LM suppresses the G1-S cell cycle progression. The G1 checkpoint, which is dependent on the p53 protein, checks for DNA damage, cell size, and growth factors [37]. The ATM/p53 pathway was activated by LM, resulting in the elevation of p21 expression (Figure 2A), a negative regulator of the cell cycle. The activity of DNA repair mechanisms is decreased by LM conditions, leading to the reduction of DNA protection against oxidative stress [38]. LM increased the production of cytosolic and mitochondrial ROS, and oxidative DNA damage (Figure 2B,C). We suggest that the LM suppresses cell cycle progression mediated by a production of ROS and DNA damage. The CDDP- and DXR-induced injuries were suppressed by LM in A549 and PC-3 cells, but not in H1299 cells, which lack endogenous p53. These results suggest that p53 plays an important role in the acquisition of chemoresistance by LM.

The development of cancer chemoresistance is upregulated by several ABC transporters including ABCB1, ABCC1, ABCC2, and ABCG2 [17]. The expression of ABCB1 was increased by LM in A549 cells (Figure 5). A variety of anticancer drugs are effluxed through ABC transporters in a substrate-selective manner. DXR, daunorubicin, and vinblastine are commonly recognized and transported by ABCB1, ABCC1, and ABCC2 [39]. The sensitivities to CDDP and DXR were attenuated by LM (Figure 3), suggesting that the efflux rates of these anticancer drugs through ABCB1 may be enhanced. In contrast, the sensitivity to ERL was unchanged by LM. ERL is a tyrosine kinase inhibitor, leading to the inhibition of tumor cell growth and synthesis of angiogenic proteins [40]. ERL has been reported to be transported through both ABCB1 and ABCG2 [41]. LM increased the expression of ABCB1, but decreased that of ABCG2. The efflux rate of ERL in LM medium may be smaller than those of CDDP and DXR. Another explanation is that ERL inhibits cell proliferation, but does not induce necrotic and apoptotic cell deaths. In contrast, CDDP and DXR induce cell death mediated by the production of ROS [42]. The difference of sensitivities to anticancer drugs may be due to the distinct action mechanisms.

Chemoresistance is associated with the existence of a sub-population of tumor cells so-called CSLCs [34]. CSLCs have the capacity for self-renewal and multilineage differentiation potential capable of generating differentiated progenitor cells. In addition, CSLCs have properties to inhibit apoptosis, and induce chemoresistance and radioresistance. LM increased the proportion of CD133-positve cells in A549 cells (Figure 8). The expression of Oct4 and Nanog, marker genes of CSLCs, was also increased by LM, which was inhibited by SB203580. We suggest that LM increases the proportion of CD133-positive cells mediated by the activation of p38 MAPK pathway. The activation of p38 is reported to be abolished by loss of p53 [40], suggesting a signaling cross-talk between p38 and p53. The activation of p38 may be also involved in the roscovitine-induced elevation of ABCB1 expression and chemoresistance. The mechanism of development of cancer stemness has not been fully understood. Melatonin reduces stemness through the inhibition of several signaling pathways including p38 MAPK in lung cancer cell lines [43]. In contrast, the p38 MAPK negatively contributes to CSLCs properties of NSCLC [44]. Further studies are needed to clarify the mechanism of promotion of cancer stemness by LM.

## 5. Conclusions

We found that LM reduces the sensitivity of A549 cells against CDDP and DXR, although it suppresses cell proliferation. The LM-induced chemoresistance was also observed in PC3 cells. Roscovitine induced cell cycle arrest in G1 phase and enhanced chemoresistance in A549 cells. LM increased the expression of ABCB1, which was inhibited by SB203580. The CDDP- and DXR-induced cell injuries were enhanced by SB203580 and PSC833. LM increased the mRNA levels of cancer stem markers and the proportion of CD133-positive A549 cells. Although the relationship between p53 and sensitivity to low Mg^2+^ has not been clarified in other cell lines, we suggest that Mg^2+^ deficiency may enhance the chemoresistance of lung adenocarcinoma and recurrence risk for lung cancer patients. Therefore, serum Mg^2+^ concentration must be strictly controlled within physiological range in the treatment of cancer patients.

## Figures and Tables

**Table 1:** Primer pairs for real-time PCR.

Genes	Direction	Sequence (5′→3′)
*TRPM6*	Sense	AAGGACTCCAGGTGCCAAT
	Antisense	TCCTCTTCAGAGATGCTGTTTTC
*TRPM7*	Sense	GCCACTTGGAAACTGGAACC
	Antisense	CGGTAGATGGCCTTCTACTG
*CNNM2*	Sense	GTTCTGGGAATCGTCACCTTAG
	Antisense	TTTCAGTTCCTGGATGACATTG
*MagT1*	Sense	GCAAACTCCTGGCGATACTCC
	Antisense	ACTGGGCTTGACTGCTTCC
*SLC41A1*	Sense	GGTCTTCATCCTAGTGCCTG
	Antisense	CAAGGTGATGAGGTCGCC
*MRS2*	Sense	GACTAATGGGAGTTGCTTTTGG
	Antisense	AATGGAGCTTCTAGCTGTCGTC
*ABCB1*	Sense	CCCATCATTGCAATAGCAGG
	Antisense	TGTTCAAACTTCTGCTCCTGA
*ABCC1*	Sense	ATGTCACGTGGAATACCAGC
	Antisense	GAAGACTGAACTCCCTTCCT
*ABCC2*	Sense	ACAGAGGCTGGTGGCAACC
	Antisense	ACCATTACCTTGTCACTGTCCATGA
*ABCG2*	Sense	AGATGGGTTTCCAAGCGTTCAT
	Antisense	CCAGTCCCAGTACGACTGTGACA
*CD133*	Sense	CGACAATGTAACTCAGCGTCTT
	Antisense	CACACAGTAAGCCCAGGTAGTA
*Oct4*	Sense	GGATCACCCTGGGATATACACA
	Antisense	TTCATTGTTGTCAGCTTCCTCC
*Nanog*	Sense	AAATGTCTTCTGCTGAGATGCC
	Antisense	CTTTGGGACTGGTGGAAGAATC
*β-Actin*	Sense	CCTGAGGCACTCTTCCAGCCTT
	Antisense	TGCGGATGTCCACGTCACACTTC

**Table 2:** Primary antibodies for Western blotting.

Name	Catalog No.	Lot No.	Supplier	Address
p-ATM (D6H9)	5883T	6	Cell Signaling Technology	Danvers, MA, USA
ATM	27156-1-AP	00055078	ProteinTech	Rosemont, IL, USA
p-p53	65415	1091	Full Moon Biosystems	Sunnyvale, CA, USA
p53 (PAb122)	MS-182-P0	182P810D	Thermo Fisher Scientific	San Jose, CA, USA
p21	14-6715	81	Thermo Fisher Scientific	San Jose, CA, USA
ABCB1	GTX108354	39834	GeneTex	Irvine, CA, USA
ABCC1	GTX116046	40135	GeneTex	Irvine, CA, USA
ABCC2	4446S	1	Cell Signaling Technology	Danvers, MA, USA
ABCG2	GTX100437	39471	GeneTex	Irvine, CA, USA
Nrf2	16396-1-AP	10898000	ProteinTech	Rosemont, IL, USA
HIF-1α	GTX113850	W	GeneTex	Irvine, CA, USA
Sp1 (1C6)	sc-420	K1907	Santa Cruz Biotechnology	Santa Cruz, CA, USA
c-Jun (60A8)	21021-1	7	Signal Antibody Technology	College Park, MD, USA
Nucleoporin p62	610497	5352647	BD Biosciences	Franklin Lakes, NJ, USA
p38	612168	15187	BD Biosciences	Franklin Lakes, NJ, USA
β-Actin	sc-1615	H3016	Santa Cruz Biotechnology	Santa Cruz, CA, USA

## Data Availability

Not applicable.
